# Synchronous Pancreatic Neoplasms Involving Pancreatic Ductal Adenocarcinoma: A Systematic Review of Case Reports

**DOI:** 10.3390/jpm15060221

**Published:** 2025-05-28

**Authors:** Daniel Paramythiotis, Eleni Karlafti, Dimitrios Tsavdaris, Alexandros Mekras, Aristeidis Ioannidis, Stavros Panidis, Elizabeth Psoma, Panos Prassopoulos, Antonios Michalopoulos

**Affiliations:** 1 First Propaedeutic Surgery Department, University General Hospital of Thessaloniki AHEPA, Aristotle University of Thessaloniki, 54636 Thessaloniki, Greece; danosprx@auth.gr (D.P.); tsavdaris@auth.gr (D.T.); ariioann@yahoo.gr (A.I.); st.panidis@gmail.com (S.P.); amichal@auth.gr (A.M.); 2 Emergency Department, University General Hospital of Thessaloniki AHEPA, Aristotle University of Thessaloniki, 54636 Thessaloniki, Greece; 3 First Propaedeutic Department of Internal Medicine, University General Hospital of Thessaloniki AHEPA, Aristotle University of Thessaloniki, 54636 Thessaloniki, Greece; 4 Department of General and Visceral Surgery, SHG-Klinikum Merzig, Academic Hospital of University of Saarland, 66663 Merzig, Germany; almekras@yahoo.gr; 5 Department of Clinical Radiology, University General Hospital of Thessaloniki AHEPA, Aristotle University of Thessaloniki, 54636 Thessaloniki, Greece; elizabethpsoma@gmail.com (E.P.); pprasopo@auth.gr (P.P.)

**Keywords:** synchronous primary tumors, pancreatic cancer, pancreatic ductal adenocarcinoma, management strategies

## Abstract

**Background:** Pancreatic ductal adenocarcinoma (PDAC) is the most common pancreatic malignancy and is characterized by a very unfavorable prognosis. Rarely, patients may develop synchronous PDAC and another distinct primary pancreatic tumor, such as a pancreatic neuroendocrine tumor. This systematic review consolidates published case reports describing the presentation, imaging characteristics, management, and outcomes of patients with synchronous PDAC and other pancreatic malignancies. **Methods**: A comprehensive search of PubMed and Scopus identified 26 relevant case reports, with inclusion criteria focused on histologically confirmed synchronous pancreatic tumors and exclusion of metastatic disease. **Results**: The majority of patients present with two pancreatic lesions, often located in both the body and tail of the pancreas. Diagnostic imaging modalities, such as computed tomography and endoscopic ultrasound, reveal common findings. Tumor markers, particularly CA 19-9, are often elevated and aid in the diagnosis. Surgical approaches also vary according to tumor location and staging, with procedures ranging from Whipple surgery to total pancreatectomy. Chemotherapy is frequently employed postoperatively. Notably, lymph node involvement and larger tumor size are associated with poorer prognoses. **Conclusions**: In conclusion, these patients may present with a common or non-common clinical picture as well as laboratory and imaging findings, constituting an important and unique diagnostic and therapeutic challenge.

## 1. Introduction

Pancreatic neoplasms, while not among the most common, have high mortality rates, placing pancreatic cancer among the five deadliest malignancies worldwide. Pancreatic ductal adenocarcinoma (PDAC), the most common type, arises from ductal cells and is aggressive, often resulting in poor clinical outcomes. Other exocrine tumors include cystic neoplasms and acinar cell carcinoma. Endocrine tumors, which originate from islet cells, range from slow-growing neuroendocrine tumors (NETs) with relatively favorable prognoses to aggressive neuroendocrine tumors with poorer outcomes [1,2,3,4,5].

Extremely rarely, two or more distinct tumors can be found in the pancreas simultaneously. The synchronous presence of multiple tumors in the pancreas may involve either independent tumors or a single lesion with mixed differentiation. The synchronous occurrence of tumors complicates differentiation from metastases and necessitates tailored diagnostic approaches [6]. Due to the rarity of such cases, no large-scale epidemiological data are currently available in the literature.

The pathogenesis of cases involving multiple invasive PDACs involves two primary mechanisms: multicentric carcinogenesis and intrapancreatic metastasis. These tumors may either arise independently at different sites within the pancreas (multicentric carcinogenesis) or develop from the spread of a primary tumor to other parts of the pancreas (intrapancreatic metastasis). In the first case, multiple cancerous lesions develop independently of each other in the pancreas. Each tumor has a separate origin and develops from a different location in the pancreatic tissue. In intrapancreatic metastasis, an initial tumor metastasizes to other parts of the pancreas. The secondary tumor originates from the primary and shares the same genetic and histological characteristics. The differentiation between multicentric carcinogenesis and intrapancreatic metastasis is important for prognosis and treatment. Evidence suggestive of multicentric carcinogenesis is the presence of pancreatic intraepithelial neoplasia (PanIN) in each tumor and the heterogeneity of immunohistochemical and/or genetic features between tumors. Evidence suggestive of intrapancreatic metastasis is the similarity of histological, immunohistochemical, and genetic features between the tumors or the absence of PanIN in the smaller tumors. Furthermore, the design of effective management strategies for synchronous pancreatic tumors is required, which will consider factors such as tumor location, size, biological behavior, and patient-specific considerations, as well as the particularities that synchronous pancreatic tumors may present [7,8].

Despite isolated case reports, there is a lack of comprehensive synthesis in the literature regarding the clinical presentation, diagnostic evaluation, and management strategies for patients with synchronous PDAC and other primary pancreatic malignancies. Therefore, the rationale for this review is to address this gap by systematically examining and summarizing all reported cases to date. This review aims to highlight the diagnostic challenges, therapeutic approaches, and outcomes in these rare but clinically significant scenarios, thereby guiding clinicians in the management of similar future cases.

## 2. Methods

### 2.1. Guidelines and Protocol

The methodology aligns with the recommendations outlined in the Cochrane Handbook for Systematic Reviews, and reporting follows the PRISMA (Preferred Reporting Items for Systematic Reviews and Meta-Analyses) guidelines to ensure transparency and methodological rigor [9,10]. The registered protocol for this systematic review can be accessed at https://doi.org/10.17605/OSF.IO/A87Q9.

### 2.2. Eligibility Criteria

We included case reports that described adult patients (≥18 years) diagnosed with synchronous pancreatic malignancies where at least one tumor was histologically confirmed as pancreatic ductal adenocarcinoma (PDAC). Eligible studies provided relevant clinical details, including diagnostic evaluation, treatment strategies, and/or outcomes. We excluded review articles, letters to the editor, editorials, commentaries, and non-English publications. Studies focused on metachronous tumors rather than synchronous primary pancreatic malignancies were also excluded. Cases with concomitant intraductal papillary mucinous neoplasms (IPMNs) and PDACs were also excluded to avoid confounding from premalignant mucinous neoplasms.

### 2.3. Search Strategy

A comprehensive literature search was conducted in PubMed and Scopus for studies published up to September 2024. Search terms included (pancreatic ductal adenocarcinoma OR PDAC) AND (synchronous neoplasms OR synchronous tumors OR synchronous lesions OR multiple primary tumors) AND (pancreas OR pancreatic neoplasms). The reference lists of included articles were manually reviewed to identify additional eligible studies. Only studies published in English were considered.

### 2.4. Study Selection

Two independent reviewers (D.T. and E.K.) screened all titles and abstracts for eligibility. The full texts of potentially relevant studies were then assessed against the inclusion and exclusion criteria. Any disagreements were resolved by consensus or through discussion with a third reviewer (D.P.).

### 2.5. Data Extraction

Data were extracted independently by two reviewers (D.T. and E.K.) using a standardized data extraction form. Discrepancies were resolved by a third reviewer (D.P.). The following information was extracted:

- Study characteristics: Authors, year of publication, country, and type of publication (case report or case series).
- Patient characteristics: Age, sex, presenting symptoms, and clinical background.
- Tumor characteristics: Tumor types, anatomical locations, size, imaging features, and tumor marker levels.
- Diagnostic tools: Imaging modalities used (e.g., CT, MRI, or EUS) and histological confirmation.
- Treatment approaches: Type of surgical intervention and the use of adjuvant or neoadjuvant therapy.
- Outcomes: Recurrence, survival status, and follow-up duration.

### 2.6. Risk of Bias Assessment

The completeness of reporting was assessed using an adapted version of the CARE (CAse REport) guidelines checklist to evaluate the quality and transparency of each included study.

## 3. Results

### 3.1. Study Selection

**Figure 1:**
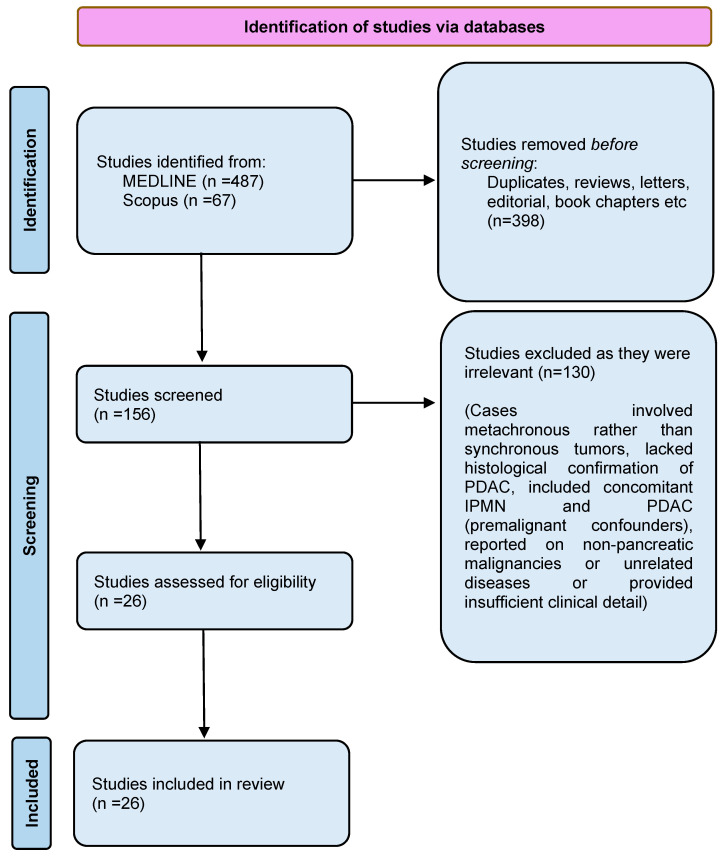
Prisma flowchart.

After searching two databases, a total of 554 studies were identified (Figure 1). After eliminating 398 studies, the remaining 156 studies were screened. During this screening process, 130 articles were excluded because they did not meet the pre-specified inclusion criteria. Finally, 26 case reports met all specified criteria and were included in this systematic review.

### 3.2. Summary of Reported Cases

We identified a total of 26 case reports in the existing literature [6,7,11,12,13,14,15,16,17,18,19,20,21,22,23,24,25,26,27,28,29,30,31,32,33,34], in which 34 cases of contemporary pancreatic tumors were analyzed, with at least one PDAC. In a pooled analysis, patients ranged in age from their forties to eighties, with a mean age of around 66 years; the sex distribution was nearly equal, with eighteen men and sixteen women affected. Notably, almost one-third of patients had a history of type 2 diabetes mellitus and nearly one-quarter had hypertension—underscoring the role of metabolic syndrome and chronic inflammatory states in the pathogenesis of pancreatic tumors. A smaller proportion had antecedent non-pancreatic malignancies or genetic syndromes, such as breast or vulvar cancer, chronic hepatitis C, or MEN-1.

Clinically, more than seventy percent of patients presented with so-called alarm symptoms—epigastric pain, weight loss, or jaundice—although these manifestations are non-specific and common to many pancreatic pathologies. A minority experienced less typical complaints, such as back pain, cholangitis, diarrhea, vomiting, or pruritus. Because these overlapping presentations seldom point to more than one lesion, synchronous tumors often escape detection until surgical exploration or postoperative histopathology. These symptoms are presented in Table 1.

Computed tomography remains the most widely used option, used in approximately two-thirds of cases, but may miss small or non-enhancing synchronous lesions. Magnetic resonance imaging (MRI) and endoscopic ultrasound (EUS) were used less frequently, in twenty-nine and twenty-one percent of cases, respectively, while MRCP (MR Cholangiopancreatography), ERCP, transabdominal ultrasound, and PET-CT were all employed in fewer than twenty percent of patients. Despite modern cross-sectional techniques, preoperative identification of both lesions was achieved in about two-thirds of the cases. When employed, EUS with fine-needle aspiration or biopsy provided crucial histological confirmation in ten cases, demonstrating that tissue sampling can avert unexpected findings at the time of surgery.

Laboratory tests have not proven effective in distinguishing between single and multiple pancreatic neoplasms. Elevated bilirubin, aminotransferases, γ-glutamyl transpeptidase, alkaline phosphatase, amylase, or glucose levels were observed only sporadically, and in many patients all values remained within normal limits. Thus, standard blood work often reflects biliary obstruction or metabolic comorbidities without signaling the presence of more than one tumor.

Preoperative biopsy practices varied widely: over half of patients underwent resection without any tissue sampling of the synchronous lesion, leading to unanticipated histologies on final pathology. EUS-guided fine-needle aspiration or biopsy accounted for only twenty-one percent of cases, while CT-guided needle biopsy, ERCP cytology, and brushings comprised the remainder. These data emphasize the need for a protocol that mandates sampling of each suspicious lesion to inform surgical planning.

Histologically, true “double PDAC”—two foci of ductal adenocarcinoma—constituted the majority of synchronous cases (59 percent), as presented in Table 2. Pancreatic neuroendocrine tumors accounted for twenty-one percent of combinations, followed by microcystic adenomas, serous cystadenomas, and rare presentations of intraductal papillary mucinous neoplasm with neuroendocrine tumor or pheochromocytoma. The heterogeneity of synchronous lesions demands an open diagnostic approach: a cystic lesion may not be a benign serous cystadenoma, and a small solid lesion may represent an aggressive adenocarcinoma rather than an indolent neuroendocrine tumor.

From a pathophysiological standpoint, the co-occurrence of PDAC with metabolic risk factors such as diabetes and hypertension suggests a shared etiologic environment characterized by chronic inflammation, insulin resistance, and perhaps a field-defect carcinogenesis within the pancreatic parenchyma. Distinguishing whether synchronous neoplasms arise independently or share molecular drivers requires further genomic and epigenetic profiling. Details of the findings are summarized and displayed in Table 3.

### 3.3. Tumor Characteristics

In the thirty-four cases of synchronous pancreatic neoplasms reviewed, the anatomical distribution of lesions was heterogeneous. Lesions confined to the pancreatic head alone accounted for 14.7 percent (5/34) of cases, whereas the body alone and the tail alone were each the sole location in only 5.9 (2/34) and 2.9 percent (1/34) of patients, respectively. In contrast, combinations involving two regions predominated: synchronous involvement of the body and tail was the most frequent pattern, observed in 35.3 percent (12/34) of cases, followed by head-and-tail lesions in 26.5 percent (9/34). Less common were head-and-body pairs (5.9 percent, 2/34) and the rare instance of two separate lesions both in the body (2.9 percent, 1/34). Lesions spanning all three regions (head, body, and tail) comprised 8.8 percent (3/34) of reports. This anatomical spectrum underscores that while isolated head lesions are conceivable, the vast majority of synchronous tumors present in multiple regions.

Tumor marker profiles revealed that a normal marker panel was noted in only 23.5 percent (8/34) of cases, whereas one or more markers—most commonly CA 19-9—were elevated in 38.2 percent (13/34). In the remaining 38.2 percent (13/34) of patients, marker data were either unreported or unavailable. Among those with elevations, CA 19-9 was the predominant biomarker, but rarer elevations (e.g., SPAN-1, sIL-2R, CEA, or CA 72-4) were documented in a handful of patients. Although elevations clearly flagged malignant behavior in many cases, the fact that nearly a quarter of patients had normal markers means that reliance on serologic surveillance alone may miss pancreatic lesions.

Tumor size varied widely. When considering the largest lesion in each patient, diameters ranged from a microscopic 1 mm up to 90 mm, with a mean maximal diameter of approximately 36 mm across thirty-two evaluable cases (range 1–90 mm). Two-thirds of cases (≈67 percent) harbored at least one lesion exceeding 30 mm. Such variability in size underscores the necessity of high-resolution imaging—smaller lesions may evade standard cross-sectional modalities, while larger masses are more readily apparent yet portend a more advanced disease state.

Lymph node status, reported in twenty-four studies, revealed that 32.4 percent (10/34) of patients had pathologically confirmed node-positive disease, whereas 8.8 percent (3/34) were explicitly node-negative. In the remaining 58.8 percent (20/34), nodal status was either not assessed preoperatively or not reported. The fact that nearly one-third of patients presented with nodal metastases highlights the aggressive nature of synchronous lesions—particularly those involving ductal adenocarcinoma—and argues for meticulous nodal evaluation, both radiographically and surgically.

Taken together, these statistics illuminate several key clinical insights. First, the predominance of body-and-tail and head-and-tail lesion pairs (together accounting for over 60 percent of cases) suggests that when a lesion is identified in one of these regions, careful scrutiny of the contralateral region is warranted. Second, although elevated CA 19-9 and other markers correlate with malignant disease in many patients, normal biomarkers cannot reliably exclude tumors. Third, the broad size distribution underscores the complementary roles of CT, MRI, and endoscopic ultrasound: while CT may readily detect larger masses, EUS is essential for evaluating and sampling smaller or equivocal lesions. Finally, the relatively high rate of nodal positivity reinforces the need for comprehensive staging, including fine-needle aspiration of suspicious nodes and consideration of extended lymphadenectomy during pancreatectomy. All relevant results are consolidated and illustrated in Table 4 and Table 5.

### 3.4. Imaging Findings

Various imaging methods were utilized in these cases to document the diagnosis, such as CT, MRI, MRCP, ERCP, and EUS.

On CT, the majority of patients showed hypodense or hypovascular masses in the pancreas, suggestive of malignant lesions. In some cases, such as those described by Ohno et al. [16], multiple masses were found in different parts of the pancreas, while in other cases findings such as mass invasion, dilated bile ducts, and dilated pancreatic ducts were found, which further indicated an advanced disease state. The two most common imaging findings were dilatation of the main pancreatic duct (MPD) and hypoattenuating masses.

MRI, in the majority of cases, confirmed the CT findings, showing poorly or heterogeneously enhancing masses. Several cases reported mass dilatation in either the intrahepatic or pancreatic bile ducts. Hypovascular or heterogeneously enhancing lesions were also reported in some cases, while other cases, such as Liu et al. [29] and Wang et al. [22], presented with a multilocular cyst. Finally, in cases like Aloraini et al., MRI demonstrated severe dilation in both the intra- and extrahepatic bile ducts [33].

EUS provided further precision in identifying hypoechoic or hypovascular lesions in many of the cases, supporting the malignancy diagnosis. In fact, hypoechoic masses were a common finding in synchronous PDAC cases. In other cases, the lesions were associated with atrophy of the surrounding pancreatic parenchyma, indicating the destructive nature of the disease. MRCP was used selectively in these case reports and provided valuable insights, especially concerning the dilation of the pancreatic and bile ducts. The presence of multiple small cystic lesions in several reports further supports the existence of synchronous tumor growth and potentially mixed pathology, such as the coexistence of cystic and solid components. ERCP findings in several cases revealed strictures or obstructions in the pancreatic and bile ducts. Nitta et al. [15] described narrowing in the MPD in both the head and body of the pancreas, indicative of multiple synchronous lesions affecting different portions of the ductal system, while Siassi et al. [14] reported obstruction of the papillary opening of the ampulla of Vater, emphasizing tumor-induced obstructions. The findings of the studies are presented in Table 6.

**Figure 2:**
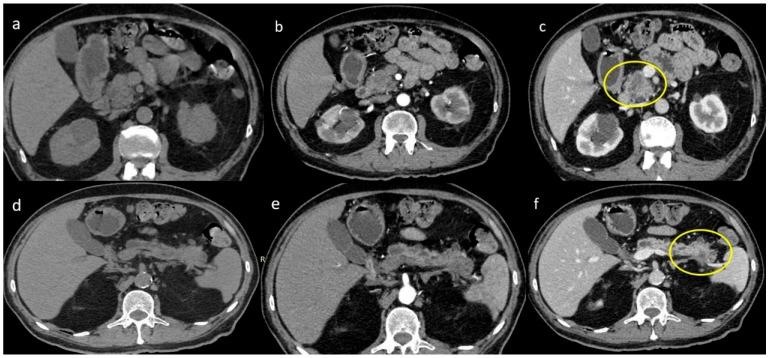
Patient with synchronous ductal adenocarcinoma of the pancreas. (**a**,**d**) NECT synchronous hypodense poorly defined masses at the head and tail of the pancreas with multiple cystic lesions in the latter and dilatation of the main pancreatic duct. (**b**,**e**) Arterial and (**c**,**f**) venous phase: the masses appear hypodense with surrounding desmoplastic reaction and infiltration to adjacent fat (Yellow circles).

**Figure 3:**
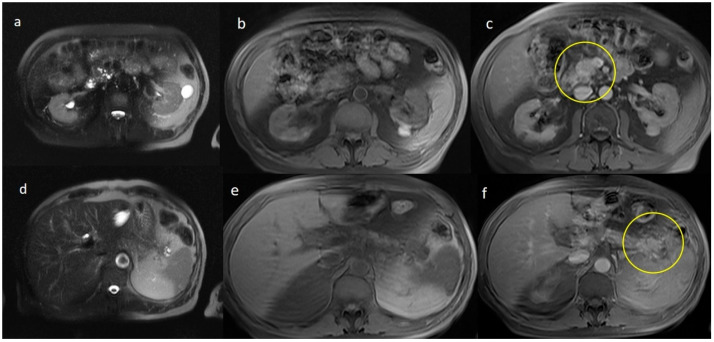
Imaging findings in the same patient with synchronous pancreatic ductal adenocarcinoma as depicted in Figure 1. (**a**,**d**) MRI T2WI with fat saturation: lobulated masses displaying intermediate signal intensity and ill-defined borders. (**b**,**e**) T1WI with fat saturation and (**c**,**f**) T1 with gadolinium and contrast enhancement (venous phase) showing inhomogenous enhancement of the masses and fat stranding (Yellow circles).

**Figure 4:**
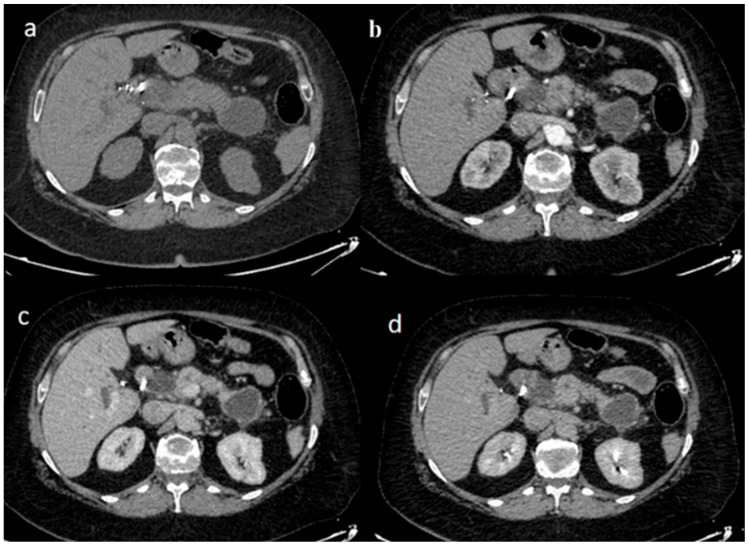
Patient with three synchronous pancreatic lesions (PDAC, IPMN, and pseudocyst). (**a**) NECT synchronous hypodense lesions at the head and tail of the pancreas with a thin wall and a less thin wall with septation, accompanied by dilation of the common bile duct. (**b**) Arterial (**c**) venous phase and (**d**) delayed phase: the masses exhibit a cystic appearance with thin wall enhancement and limited fat stranding.

Three imaging features of synchronous malignancies of the pancreas are exposed on CT and MRI in Figure 2, Figure 3 and Figure 4. Figure 2 demonstrates NECT images, showing synchronous hypodense, ill-defined masses located in both the head and tail of the pancreas, the latter associated with multiple small cystic lesions and a dilated main pancreatic duct. The arterial (b) and venous (c) phases further reveal those masses as hypodense, associated with desmoplastic reaction surrounding the mass, along with adjacent fat infiltration, conforming with reports on malignancies (PDAC). Figure 3 depicts MRI findings on T2-weighted imaging (T2WI), pointing out to the lobulated masses of intermediate signals and irregular borders. T1-weighted imaging (T1WI) and T1-weighted GD-venous phases reveal inhomogeneous enhancement and fat stranding around the masses. Lastly, Figure 4 shows NECT and contrast-enhanced images of synchronous hypodense lesions in the head and tail of the pancreas, revealing cystic variety, enhanced by a thin wall, associated with common bile duct dilation and mild fat stranding. Altogether, these figures emphasize various imaging characteristics of synchronous pancreatic malignancies.

### 3.5. Management of Synchronous Pancreatic Cancers

The anatomical distribution of synchronous pancreatic neoplasms in this thirty-four-patient series carries profound implications for both operative planning and long-term patient management. Although isolated lesions confined to the head, body, or tail represent only a minority of cases—14.7 percent, 5.9 percent, and 2.9 percent, respectively—more than two-thirds of patients harbored tumors spanning two or more regions. Lesions involving the body and tail predominated (35.3 percent), closely followed by combined head and tail involvement (26.5 percent). Rarer patterns included head-and-body tumors (5.9 percent) and multifocal disease across all three regions (8.8 percent)

These distributions directly inform the choice of resection and the balance between oncologic adequacy and preservation of pancreatic function. When synchronous tumors reside exclusively within the pancreatic head, the Whipple procedure or pylorus-preserving pancreaticoduodenectomy remains the standard, ensuring wide margins. By contrast, neoplasms confined to the tail are amenable to distal pancreatectomy, often with spleen preservation if oncologically safe.

In the far more common scenario of body-and-tail involvement—seen in over one-third of patients—surgeons face a choice between extended distal resection and total pancreatectomy. Extended distal pancreatectomy may preserve head function but risks leaving residual tumor in the uncinate process. Conversely, total pancreatectomy guarantees complete removal of multifocal disease but induces surgically-induced diabetes and exocrine insufficiency in all patients. Given that two-region involvement (body + tail and head + tail) comprises more than 60 percent of synchronous cases, the need to weigh endocrine and exocrine sequelae against oncologic safety is paramount. In younger patients with robust metabolic reserve, total pancreatectomy may be justified; in older or frailer patients, parenchyma-sparing alternatives combined with vigilant surveillance of the remnant may strike a more appropriate balance.

Head-and-tail involvement poses its own unique challenges. A combined Whipple and distal resection (middle segment preservation pancreatectomy) can leave only a small pancreatic segment in the body, which often lacks sufficient vascularization and exocrine function. Many centers therefore choose total pancreatectomy in such cases to avoid the high risk of postoperative fistula and residual disease. This approach requires preoperative counseling of the patient regarding lifelong insulin dependence and pancreatic enzyme replacement.

Finally, the presence of synchronous disease in all three regions (8.8 percent) essentially mandates total pancreatectomy. Although this gives the best chance for oncologic clearance, the trade-off is permanent endocrine and exocrine deficiency. Such patients benefit from multidisciplinary pre- and postoperative management, including endocrinology consultation for intensive insulin therapy and gastroenterology support for pancreatic enzyme replacement.

Across all patterns, close collaboration between surgical, oncology, endocrinology, and nutrition teams is critical. Preoperative assessment must include high-resolution imaging to delineate each lesion’s exact location and relationship to vital structures. Intraoperative frozen sections can verify margins and guide the extent of resection. Post-resection, adjuvant chemotherapy regimens—typically gemcitabine- or FOLFIRINOX-based—should be tailored to the dominant histology, often PDAC, but with consideration of any neuroendocrine or cystic components. Lifelong follow-up with imaging and tumor markers is essential, as the residual pancreas (if any) remains at risk for metachronous lesions.

In summary, the anatomical patterns of synchronous pancreatic tumors demand a management strategy that systematically balances oncologic completeness with preservation of endocrine and exocrine function. For lesions confined to a single region, standard resections suffice. When two or more regions are involved—accounting for the majority of cases—surgeons must carefully weigh extended or total pancreatectomy against the metabolic consequences, deploying parenchyma-sparing techniques only in highly selected scenarios. This nuanced, location-driven approach offers the best path to optimize both survival and quality of life in this challenging patient population. The findings of the studies are presented in Table 7.

### 3.6. Risk of Bias

Overall, the thirty-four case reports demonstrated high compliance with the CARE guidelines, with clear documentation of clinical presentation, diagnostic workup, treatment, and follow-up. Most included structured abstracts and comprehensive discussions, allowing for reliable interpretation and comparison. The only consistent limitation was the absence of the patient’s perspective across all reports, which restricts insight into the personal and quality-of-life impacts of managing synchronous pancreatic neoplasms. Including the patient voice in future reports would enhance the depth and applicability of clinical evidence.

## 4. Discussion

### 4.1. Diagnostic Challenges

In the existing literature there are only 26 case reports and no other original articles concerning synchronous pancreatic malignancies with PDAC. In this review, the results of these case reports are analyzed and conclusions are given regarding both the diagnosis and the management of these patients. These results reveal the high heterogeneity among concurrent pancreatic tumors. This heterogeneity concerns all aspects of the tumor, i.e., both the clinical manifestation of the patients, as well as the laboratory and imaging findings. It is also understood through these results that synchronous pancreatic tumors can occur in any anatomical region of the pancreas, while more than two lesions can also appear. Most patients presented with two pancreatic lesions, commonly located in both the head and tail of the pancreas. Additionally, another common finding is elevated tumor markers, particularly CA 19-9, while the absence of tumor markers does not exclude the diagnosis. Tumor sizes varied widely, with lesions ranging from 5 mm to over 65 mm. Several patients exhibited involvement of lymph nodes, suggesting a more advanced stage of disease, with some showing multiple metastases, indicative of poorer prognosis.

### 4.2. Differential Diagnosis and Imaging Characteristics

The differential diagnosis of synchronous multiple lesions of the pancreas includes many clinical entities. These include both benign and malignant diseases. Specifically, benign diseases include a large percentage of patients with synchronous multiple lesions of the pancreas, with autoimmune pancreatitis (AIP) as the main cause. Specifically, approximately 1 in 5 patients with AIP have multifocal locations within the pancreas. Evidence shows that multifocal AIP may be associated with inflammatory bowel disease, while in these patients, higher serum IgG4 levels, multiple areas of smooth stenosis of the MPD, a capsule-like rim around the pancreas, and lower apparent diffusion coefficient are observed compared to PDAC patients [35,36,37,38]. Malignant diseases that must be differentiated from the existence of synchronous multiple tumors of the pancreas include both the malignancies that being described in this review, as well as pancreatic neuroendocrine tumors in multiple endocrine neoplasia type 1, metastases, primary pancreatic lymphoma that is classified as a non-Hodgkin’s lymphoma arising from an extranodal site, as well and renal cell carcinoma and pancreatic sarcoidosis [39,40,41,42]. Distinguishing synchronous PDAC from AIP or NETs relies on subtle imaging findings. AIP typically shows a capsule-like rim with smooth MPD stenoses and elevated IgG4, whereas PDAC presents as an irregular hypovascular mass with abrupt duct cutoff. NETs often enhance hypervascularly on arterial-phase CT/MRI, in contrast to the hypoattenuating pattern of PDAC. Incorporation of diffusion-weighted MRI or contrast-enhanced EUS may further refine preoperative characterization [43,44].

In these 26 case reports, 18 cases of synchronous primary PDAC, seven cases of coexistence of PDAC and NET, one case of ampulla tumor and PDAC, one case of PDAC, IPMN, and NET, and one case of PDAC and PGL are described. The 18 cases of synchronous primary PDAC occur without evidence of metastatic spread, thus making it difficult to distinguish them from metastases. Patients with synchronous primary PDAC usually range in age from 60 to 70 years, consistent with the general population affected by PDAC. Clinically, these patients present with common symptoms such as abdominal pain, jaundice, and weight loss, although a significant number of cases are asymptomatic and the tumors are found incidentally during imaging for other causes. CT often shows hypodense masses, while MRI shows hypovascular lesions accompanied by dilatation of the MPD. EUS may visualize hypoechoic or hypovascular masses. The characteristics of these tumors are varied, with lesion sizes ranging from 5 mm to more than 60 mm. Tumor markers, particularly CA 19-9, are often elevated, helping the diagnosis, although this is not generally consistent, and in some patients, other markers such as CEA or SPAN-1 may be elevated. Lymph node metastasis is observed in several cases, signaling an advanced stage and often associated with a worse prognosis. Depending on the location of the tumor, surgical procedures such as the Whipple procedure, total pancreatectomy, or peripheral pancreatectomy are usually performed. Postoperative chemotherapy, usually with regimens such as gemcitabine or FOLFIRINOX, is often used, particularly in cases with larger tumors or lymph node involvement.

In cases where both PDAC and NETs coexist, the situation is complicated by the different biological behaviors of these tumors. PDAC is aggressive and fast growing, while NETs are slower growing. Patients with these double tumors may experience the typical symptoms, such as jaundice and abdominal pain, but also more atypical ones such as diarrhea and vomiting, depending on the behavior of the NET. Imaging findings in patients with PDAC and NET often suggest the different nature of these tumors. PDAC usually presents as hypodense, poorly enhancing masses, whereas NET may present as hypervascular or cystic lesions. CT and MRI are often used to visualize these differences, with multiple lesions often found in different areas of the pancreas. EUS also has an important role in diagnosis, detecting both hypoechoic lesions characteristic of PDAC and vascular or cystic lesions of NET. In some cases, dilatation of the bile duct or pancreatic duct may indicate advanced disease. The tumors in these cases vary considerably in size, with NET lesions often being larger and less invasive compared to PDAC. Tumor markers such as CA 19-9 are often elevated in the presence of PDAC, while specific NET markers such as chromogranin A may help diagnose the NET component. The coexistence of PDAC and NETs belongs to the category of coexistence of two different types of neoplasms within the pancreas defined as concomitant or collision tumors. These are rare entities, where the diagnosis is usually made postoperatively due to the lack of preoperative pathognomonic features [45]. However, the existence of mixed adenoneuroendocrine carcinoma of the pancreas is also possible, as described by Mori et al. [24]

Five cases of serous cystadenoma (SCA) coexisting with PDAC are also described in four available case reports [11,13,19,32]. Serous cystadenoma is a benign, cystic tumor of the pancreas, typically composed of numerous small, fluid-filled cysts. It is most commonly found incidentally during imaging, as it usually does not cause symptoms unless it grows large enough to compress nearby structures. Unlike other cystic pancreatic neoplasms, such as mucinous cystic neoplasms, serous cystadenomas have little to no malignant potential. These tumors predominantly affect middle-aged to elderly women and are generally managed conservatively unless symptomatic or very large [46,47]. Very rarely, these neoplasms coexist with PDAC. Montag et al. suggested that the coexistence of these neoplasms may be due to an abnormality in the exocrine portion of the pancreas, as in both types of neoplasms the loss of part of the 10q chromosome is observed [12].

The clinical picture of patients depends significantly on the localization of the tumor in the pancreas. In particular, in PDACs that appear in the head of the pancreas, the most common symptom is jaundice, while tail tumors usually appear with abdominal pain [48,49]. In synchronous pancreatic malignancies, these symptoms can coexist due to the simultaneous location of the tumors in more than one location. Additionally, the prognosis of the patients seems to be affected by the location of the tumor in the pancreas, with tumors in the head of the pancreas having a longer survival as more intense symptoms lead to earlier diagnosis, while tail tumors are characterized by fewer positive lymph nodes [50,51]. In concurrent pancreatic tumors, these tumor site–survival relationships may be affected by multiple tumor sites.

Overall, patients with synchronous primary PDAC or coexisting PDAC and NET present unique diagnostic and therapeutic challenges. For this reason, it is extremely important to emphasize how the management of these tumors differs from, as well as shares common characteristics with, the available guidelines for pancreatic cancer.

In particular, the diagnosis of synchronous pancreatic tumors does not differ from the diagnostic process of pancreatic cancer. According to the systematic review guidelines for the diagnosis of pancreatic cancer by Liu et al. [52], only four of the nine recent guidelines had an overall score of more than 60% and are recommended for clinical use [53,54,55,56]. Based on the guidelines evaluated in this review, the diagnostic process for pancreatic cancer should begin with history taking and physical examination, paying particular attention to symptoms and signs such as abdominal pain, acute pancreatitis, nausea, vomiting, weight loss, steatorrhea, anorexia, recent onset or exacerbation of diabetes, obstructive jaundice, and palpable mass. Then follow the laboratory and imaging tests. In terms of laboratory and imaging findings, these share the same principles as solitary PDAC. In particular, CA 19-9 is one tumor marker for PDAC, although it is characterized by low diagnostic accuracy. Its use is usually helpful in diagnosis, when associated with appropriate imaging methods. In diagnosis, it seems that the indicators CA242 and CEA may also be useful, but also with low diagnostic accuracy, especially the latter [57,58]. The imaging methods include both CT and MRI, as well as ERCP and EUS. In fact, the last two options provide extra therapeutic and diagnostic possibilities, with ERCP being able to be used to relieve biliary or pancreatic duct obstructions caused by tumors and EUS enabling tissue sampling through biopsies. However, the invasive nature of these two techniques naturally hides complications such as bleeding or tumor seeding [59,60,61].

The association of intraductal papillary mucinous neoplasms (IPMN) and PDAC is well established, as IPMNs can be considered a precursor lesion to PDAC [6]. Specifically, some IPMNs, particularly those involving the MPD (main duct IPMN), can undergo malignant transformation, eventually leading to PDAC. This progression happens over time as genetic mutations accumulate. There are many cases of coexistence of IPMN and NET pancreatic cancers available in the literature [62,63,64,65,66,67,68,69,70,71,72,73]. Therefore, because IPMN has a more favorable prognosis than PDAC, particularly when detected early and treated before invasive cancer develops, it is worth mentioning that the early diagnosis of these cases can significantly contribute to the prognosis of patients with synchronous pancreatic tumors. In the cases of coexistence of IPMN and NET, it seems that these are usually low-grade neoplasms of small size that often manifest with abdominal or back pain, or even asymptomatically, and usually occur in female patients. The association between the two types of neoplasms is covered by the term mixed neuroendocrine non-neuroendocrine neoplasms [45], and therefore in patients with IPMN, in addition to investigating the existence of extrapancreatic malignancy with which it often coexists, it is also worth investigating the existence of NET. IPMN can also coexist with other pancreatic neoplasms such as solid pseudopapillary neoplasm, a rare and low-grade neoplasm with low potential for malignancy [74,75].

### 4.3. Treatment Approaches and Surgical Strategies

However, regarding the management of synchronous pancreatic tumors, differences are observed, which are mainly due to the multiple location of the tumor. In particular, when the tumor is located in the head of the pancreas, then the Whipple procedure is preferred, which includes the removal of the head of the pancreas, the duodenum, part of the stomach, the gallbladder and the common bile duct. Conversely, when the tumor is located in the body or tail of the pancreas, then distal pancreatectomy is chosen. Laparoscopic and robotic techniques are increasingly used for pancreatectomies. In contrast to synchronous pancreatic tumors, the multiple localization of the tumors complicates the choice. The most common option is total pancreatectomy, while in some cases the Whipple procedure and distal pancreatectomy can also be chosen. Total pancreatectomy provides a more comprehensive treatment, ensuring that no microscopic disease is left behind. The choice of procedure ultimately depends on the location, size, and extent of the tumors, as well as the patient’s overall health and ability to tolerate more extensive surgery [53,54,55,56,76,77,78,79,80,81]. Chemotherapy follows the same principles as for single tumors, with FOLFIRINOX being the optimal choice and gemcitabine and chemoradiotherapy also being alternatives [82,83]. Therefore, total pancreatectomy is considered the treatment of choice in multiple synchronous pancreatic tumors in the head and tail. However, this is a surgical option that carries multiple complications [84]. These concern both the endocrine and exocrine part of the pancreas, while the 30-day mortality rate of the operation is around 5% [85]. The most important metabolic complication of the operation is diabetes, characterized as type 3c diabetes or pancreatogenic diabetes. The lack of insulin production and glucagon secretion makes glycemic control difficult in this form of diabetes, which gives it the name “brittle” diabetes [86]. However, these patients can be managed satisfactorily and in fact patients who underwent total pancreatectomy due to malignancy seem to be managed more effectively than patients who underwent surgery due to chronic pancreatitis [87]. Complications in patients in whom invasive lesions are identified in only a single region of the pancreas can be prevented by middle-preserving pancreatectomy; however, this option cannot be easily adopted in patients with multiple locations of the tumor, making the management of complications a particularly difficult challenge [88].

Outcome data for synchronous PDAC remain scant. In our pooled series, median survival after resection was approximately 12 months, compared to ~18 months for solitary resected PDAC in contemporary cohorts. Patients with dual PDAC foci exhibited a higher rate of nodal positivity (≈32 percent) and shorter disease-free intervals than those with PDAC + NET combinations, suggesting that pure ductal synchrony portends a worse prognosis. Prospective registries should stratify survival by histologic pairing to validate these observations. Regarding the prognosis of patients with PDAC, the most important factor that determines the outcome in patients is the surgical removal of the tumor. Neoadjuvant therapy may improve the likelihood of a negative surgical margin, which is associated with improved survival. Other prognostic markers include age, tumor stage, presence of lymph node metastasis, histological differentiation (with poorly differentiated tumors having a worse prognosis), tumor size (with tumors larger than 2 cm in diameter having a worse prognosis), and the presence of vascular infiltration [89,90,91]. In addition, the most important prognostic indicators for PanNETs are tumor grade and stage, while other prognostic indicators are also age, gender (with men having a slightly worse prognosis), the presence of symptoms at diagnosis, the presence of metastasis, surgical removal, chemotherapy, hormone therapy, radiation therapy, and certain molecular markers such as mutations in the KRAS and DAXX/ATRX genes [86,92,93,94].

### 4.4. Clinical Implications and Future Directions

The results of this systematic review highlight the urgent need for a more nuanced, multidisciplinary approach to the management of modern pancreatic neoplasms: clinicians should adopt dual imaging (CT or contrast-enhanced MRI plus endoscopic ultrasound) enhanced with targeted fine-needle aspiration of all suspicious lesions to ensure early detection and accurate histological characterization. Surgical planning should be individualized according to the distribution of the lesion, balancing oncological clearance through total or segment-sparing pancreatectomy against the risks of endocrine and exocrine insufficiency with preventive nutritional and diabetic support. Comprehensive molecular profiling of each tumor—including assessment of KRAS, TP53, BRCA2, PALB2, and CDKN2A status—can differentiate independent primary tumors from intrapancreatic spread, inform adjuvant treatment options (e.g., consideration of modified FOLFIRINOX or PARP inhibitors in cases with germline mutations), and guide familial risk assessment. Finally, given the higher rates of lymph node positivity and shorter disease-free intervals seen in contemporary ductal presentations, surveillance protocols should be intensified (e.g., quarterly imaging in the first postoperative year) and supported by multidisciplinary tumor boards, while professional associations work to achieve consensus on guidelines codifying best practices in imaging, surgical strategy, molecular testing, adjuvant therapy, and follow-up specifically for this complex patient population.

Understanding the molecular and genetic pathways that distinguish synchronous PDAC from metastatic disease should be the main goal of future research on synchronous PDAC. It is important to investigate the function of genetic abnormalities, such as those in TP53 and KRAS, to ascertain if intrapancreatic metastasis or multicentric carcinogenesis is the cause of synchronous PDAC. Large-scale research is also required to evaluate the effects of different treatment plans; specifically, how chemotherapy and surgical techniques impact these patients’ long-term survival and recurrence rates. To identify numerous lesions early and track the evolution of the disease more effectively, further research should be focus on advanced imaging techniques, such as the possible utilization of radiomics as well as the function of biomarkers such as CA 19-9. Investigating the genetic predispositions and environmental risk factors contributing to the development of multiple pancreatic neoplasms will also improve early detection and personalized treatments.

## 5. Conclusions

In conclusion, synchronous pancreatic malignancies involving PDAC are rare but present significant diagnostic and therapeutic challenges. Most patients show two lesions, commonly in the head and tail of the pancreas, with elevated CA 19-9 and distinct imaging findings aiding diagnosis. Surgical management varies, from Whipple procedures to total pancreatectomy, depending on tumor location, while chemotherapy, including gemcitabine and FOLFIRINOX, is essential postoperatively or for recurrence. Larger tumor size and lymph node involvement correlate with worse outcomes. Although uncommon, these cases demand a specialized approach to improve prognosis and treatment success. Future research should focus on molecular profiling, standardized treatment strategies, and multicenter data collection. This review may help inform clinical practice by increasing awareness and supporting more consistent diagnostic and therapeutic decision-making.

## Figures and Tables

**Table 1:** Summary of clinical presentation of the included cases.

Symptom	*n* (%)
Epigastric pain	9 (26%)
Weight loss	8 (24%)
Jaundice	7 (21%)
Abdominal discomfort	2 (6%)
Abdominal pain	2 (6%)
Back pain	1 (3%)
Cholangitis	1 (3%)
Diarrhea	1 (3%)
Vomiting	1 (3%)
Pruritus	1 (3%)

**Table 2:** Summary of diagnosis combination of the included cases. Abbreviations: PDAC (pancreatic ductal adenocarcinoma), SCA (serous cystadenoma), NET (neuroendocrine tumor), IPMN (intraductal papillary mucinous neoplasm), and PGL (pancreatic gastrointestinal stromal tumor).

Diagnosis Combination	*n* (%)
Synchronous PDAC	20 (59%)
PDAC + NET	7 (21%)
PDAC + microcystic adenoma	3 (9%)
PDAC + serous cystadenoma (SCA)	2 (6%)
PDAC + IPMN + NET	1 (3%)
PDAC + pheochromocytoma (PGL)	1 (3%)

**Table 3:** This table presents key characteristics of studies reporting cases of synchronous pancreatic malignancies. The columns include the study ID, which is a unique identifier for each study; sex, indicating the patient’s sex as F (Female) or M (Male); age, representing the patient’s age at diagnosis; symptoms, describing the clinical presentation or reported symptoms; and imagistic evaluation, outlining the imaging modalities used for diagnosis, including CT (computed tomography), EUS (endoscopic ultrasound), MRI (magnetic resonance imaging), MRCP (magnetic resonance cholangiopancreatography), and PET-CT (positron emission tomography–computed tomography). Preoperative biopsy refers to the type of biopsy performed, such as FNA (fine-needle aspiration) or FNB (fine-needle biopsy). Diagnosis specifies the final diagnosis of the pancreatic lesions, including PDAC (pancreatic ductal adenocarcinoma), SCA (serous cystadenoma), NET (neuroendocrine tumor), IPMN (intraductal papillary mucinous neoplasm), and PGL (pancreatic gastrointestinal stromal tumor).

Study ID	Sex	Age	Medical History	Symptoms	Imagistic Evaluation	Laboratory Results	Preoperative Biopsy	Diagnosis
Posniak et al., 1990 [11]	F	79	Unremarkable	Weight loss Epigastric pain	CT	-	CT-guided FNAB	PDAC and microcystic adenoma
Montag et al., 1990 [12]	M	62	-	Weight loss Epigastric pain	-	-	-	PDAC and microcystic adenoma
	F	59	-	Epigastric pain	-	-	-	PDAC and microcystic adenoma
Nodell et al., 1993 [13]	M	70	-	Jaundice Weight loss Epigastric pain	CT, ERCP	-	CT-guided FNAB	PDAC and SCA
Siassi et al., 1999 [14]	F	62	-	Jaundice	US, CT	-	CT-guided FNAB	Synchronous PDAC
Nitta et al., 2008 [15]	F	79	-	-	CT, MRI	Elevated bilirubin and aminotransferases	-	PDAC and SCA
Izumi et al., 2009 [16]	F	75	Chronic hepatitis C	Back pain	CT, MRCP, PET-CT	-	No	Synchronous PDAC
Fujimori et al., 2010 [17]	M	77	T2DM	-	CT, US	Elevated fasting glucose level and HBA1C	No	Synchronous PDAC
Mori et al., 2010 [18]	M	57	T2DM	-	US, CT, MRI, MRCP, ERCP	Unremarkable	ERCP cytology	Synchronous PDAC
Goong et al., 2015 [19]	F	61	-	Abdominal discomfort Jaundice	CT, PET-CT	Elevated bilirubin, aminotransferases, and γ-glutamyl transpeptidase	EUS-FNB	Synchronous PDAC
De Silva et al., 2017 [20]	F	65	Cholangitis	Weight loss Jaundice	CT, MRI, ERCP	Elevated bilirubin and alkaline phosphatase	Brush cytology	Synchronous PDAC
Serafini et al., 2017 [21]	F	69	Breast cancer, T2DM, hypertension, and hypercholesterolemia	Weight loss Epigastric pain	CT, EUS	-	-	PDAC and NET
Wang et al., 2018 [22]	F	51	T2DM Hypertension	Epigastric pain	CT, EUS	Unremarkable	EUS-FNA	PDAC and NET
Kim et al., 2018 [23]	M	64	Whipple operation due to an adenocarcinoma in the pancreatic head	-	CT, MRI	-	No	PDAC and NET
Mori et al., 2018 [24]	M	52	10 cigarettes per day for 30 years	Epigastric pain	CT, EUS, MRCP	Unremarkable	No	PDAC and NET
Huang et al., 2018 [25]	M	48	Basal cell carcinoma, hypertension, hyperlipidemia, and T2DM	Abdominal pain Diarrhea Vomiting Weight loss	CT	Elevated bilirubin	EUS-FNA	PDAC and NET
McGregor et al., 2018 [26]	M	72	-	-	-	-	EUS-FNB	Synchronous PDAC
Sugiura et al., 2019 [27]	F	69	-	-	CT, EUS	-	EUS-FNA	Synchronous PDAC
Ohno et al., 2020 [28]	M	54	Hyperparathyroidism (MEN-1)	-	CT, EUS		EUS-FNA	PDAC and NET
Liu et al., 2020 [29]	M	74		Epigastric pain	CT, MRI	Elevated pancreatic amylase	No	PDAC and NET
Nitta et al., 2020 [15]	F	77	-	-	CT, PET-CT	Unremarkable	EUS-FNA	Synchronous PDAC
Ohike et al., 2020 [7]	F	70		-	MRI		EUS-FNA	Synchronous PDAC
Fujita et al., 2020 [30]	M	70s		-	-		-	Synchronous PDAC
	F	70s		-	-		-	Synchronous PDAC
	M	60s		-	-		--	Synchronous PDAC
	M	60s		-	-		-	Synchronous PDAC
	M	40s		-	-		-	Synchronous PDAC
	F	70s		-	-		-	Synchronous PDAC
	M	80s		-	-		-	Synchronous PDAC
Schlanger et al., 2021 [6]	M	54	T2DM Hypertension	-	US, EUS, MRI	Unremarkable	EUS-FNA	PDAC, IPMN, and NET
Aaquist et al., 2021 [31]	F	70	Breast cancer Vulvar cancer	Jaundice Weight loss Epigastric pain	CT		No	PDAC and PGL
Paramythiotis et al., 2022 [32]	M	80	Hypertension, T2DM, dyslipidemia hyperuricemia, and hypothyroidism	Abdominal discomfort Weight loss	CT, EUS, MRI, MRCP	Elevated blood glucose levels	EUS-FNB	Synchronous PDAC
Aloraini et al., 2023 [33]	M	66	T2DM, dyslipidemia, and hypertension	Jaundice	CT, MRI	Elevated bilirubin, aminotransferases, and γ-glutamyl transpeptidase	No	Synchronous PDAC
	F	56	Asthma T2DM	Jaundice Pruritus Abdominal pain	CT, MRI, MRCP, ERCP	Elevated bilirubin and leukocytosis	No	Synchronous PDAC

**Table 4:** This table summarizes key characteristics of pancreatic tumors. The columns include the location, indicating the anatomical site of the tumor within the pancreas; number of lesions, specifying the count of distinct tumor lesions; tumor markers, listing biomarkers assessed in the study, including CA 19-9 (carbohydrate antigen 19-9), SPAN-1 (sialylated pancreatic antigen), sIL-2R (soluble Interleukin-2 Receptor), and CEA (carcinoembryonic antigen); diameter of tumor (mm), representing the size of the tumor in millimeters; and metastatic lymph nodes, detailing the involvement of lymph nodes by metastatic spread.

Study ID	Location	Number of Lesions	Tumor Markers	Diameter of Tumor (mm)	Metastatic Lymph Nodes
Posniak et al., 1990 [11]	Head and tail	2	Normal	40 (head)	-
Montag et al., 1990 [12]	Body	2	-	-	-
	Body and tail	2	-	-	-
Nodell et al., 1993 [13]	Head	2	-	80, 30	Positive
Siassi et al., 1999 [14]	Body and tail	2	Normal	10 (body), 50 (tail)	Negative
Nitta et al., 2008 [34]	Head	2	Normal	30, 30	-
Izumi et al., 2009 [16]	Head and body	4	Elevated CA 19-9, SPAN-1	25 (head), 20 (head), 10 (body), 10 (body)	Positive
Fujimori et al., 2010 [17]	Head, body, and tail	3	Elevated CA 19-9, sIL-2R	20 (head), 35 (body), 15 (tail)	Positive
Mori et al., 2010 [18]	Head and tail	2	Elevated CA 19-9	12 (head), 3 (tail)	Positive
Goong et al., 2015 [19]	Head and tail	2	Elevated CA 19-9	49 (head), 24 (tail)	Positive
De Silva et al., 2017 [20]	Head and tail	2	-	50 (tail), 30 (head)	-
Serafini et al., 2017 [21]	Head to the tail	2	Elevated CA 19-9	50 (body–tail)	Positive
Wang et al., 2018 [22]	Head	2	Normal	18	-
Kim et al., 2018 [23]	Body and tail	2	Normal	12 (body), 5 (tail)	-
Mori et al., 2018 [24]	Head	2	Elevated CA 19-9	32 (head)	-
Huang et al., 2018 [25]	Head and body	2	Elevated CA 19-9	25 (head), 19 (body)	-
McGregor et al., 2018 [26]	Head and tail	2	-	13 (head), 14 (tail)	Negative
Sugiura et al., 2019 [27]	Body and tail	2	-	35 (body), 23 (tail)	-
Ohno et al., 2020 [28]	Tail	2	Elevated CA 19-9	30 (head), 16 (tail)	Positive
Liu et al., 2020 [29]	Body and tail	2	Elevated CA 19-9	40 (body), 6 (tail)	-
Nitta et al., 2020 [15]	Head and tail	2	Normal	18 (head), 32 (tail)	-
Ohike et al., 2020 [7]	Body and tail	2	Elevated CA 19-9	19 (body), 45 (tail)	Negative
Fujita et al., 2020 [30]	Body and tail	2	-	16 (body), 29 (tail)	-
	Body and tail	2	-	20 (body), 30 (tail)	-
	Body and tail	2	-	8 (body), 24 (tail)	-
	Body and body	2	-	12 (body), 1 (body)	-
	Body and tail	2	-	35 (body), 1 (tail)	-
	Body and tail	2	-	32 (body), 1 (tail)	-
	Body and tail	2	-	7 (body), 30 (tail)	-
Schlanger et al., 2021 [6]	Head and tail	3	Normal	90 (head), 7 (head), 6 (tail)	-
Aaquist et al., 2021 [31]	Head	2	Normal	18 (head), 13 (head)	-
Paramythiotis et al., 2022 [32]	Head and tail	2	Elevated CA 19-9, CA 72-4, normal CEA	35 (head), 45 (tail)	Positive
Aloraini et al., 2023 [33]	Head, body, and tail	Multiple	Elevated CEA, CA 19-9	65 (head)	Positive
	Head, body, and tail	Multiple	Elevated CEA, CA 19-9	50 (head)	Positive

**Table 5:** Location Pattern of the included cases.

Location Pattern	Number of Cases (n)	Percentage (%)
Head alone	5	14.7
Body alone	2	5.9
Tail alone	1	2.9
Body + tail	12	35.3
Head + tail	9	26.5
Head + body	2	5.9
Head + body + tail	3	8.8

**Table 6:** This table presents findings from various imaging modalities used in the evaluation of pancreatic tumors. The columns include CT (computed tomography), MRI (magnetic resonance imaging), MRCP (magnetic resonance cholangiopancreatography), ERCP (endoscopic retrograde cholangiopancreatography), and EUS (endoscopic ultrasound). Each column outlines the specific observations and diagnostic details identified by the respective imaging techniques. SMV: superior mesenteric vein, pp: pancreatic parenchyma.

Study ID	CT	MRI	MRCP	ERCP	EUS
Posniak et al., 1990 [11]	Well-defined in the head and in the tail	-	-	-	-
Montag et al., 1990 [12]	Low- attenuation mass in the head	-	-	MPD obstruction	-
Siassi et al., 1999 [14]	Dilated nitre- and extrahepatic bile ducts	-	-	MPD splitting in the tail region and obstruction near the ampulla of Vater	-
Nodell et al., 1993 [13]	Cystic mass and an identical-sized solid mass adjoining in the head	Cystic mass and an identical-sized solid mass adjoining in the head	-	-	-
Siassi et al., 1999 [14]	Hypodense masses	-	MPD dilation and stenosis	MPD stenosis	-
Nitta et al., 2008 [34]	Hypovascular masses in the head and body MPD dilation	-	Narrowing of the MPD in the head and body	Stricture of the lower bile duct without dilatation of the upstream bile duct	Hypoechoic lesions MPD dilation
Izumi et al., 2009 [16]	Hypodense mass in tail	-	Two small cystic lesions	MPD dilation	-
Fujimori et al., 2010 [17]	Mass in the head with duodenal invasion	-	-	-	Hypoechoic lesions
Mori et al., 2010 [18]	Ampullary tumor with ill-defined margins invading adjacent pancreatic tissue and PDAC with irregular margins	Dilated CBD with smooth tapering stricture at the distal end and the ampullary tumor inferior to the distal end of CBD	-	-	-
Goong et al., 2015 [19]	MPD dilation	-	-	-	Hypoechoic lesions MPD dilation
De Silva et al., 2017 [20]	Well-circumscribed, heterogeneously enhancing lesion in the head	-	-	-	Well-demarcated hypoechoic mass
Serafini et al., 2017 [21]	MPD dilation Enhanced nodule	Poorly enhanced lesion in the body	-	-	-
Wang et al., 2018 [22]	MPD dilation Multilocular cysts in the head	-	MPD dilation Multilocular cysts in the head	Dilation of the papillary opening of Vater	MPD in the head was surrounded by multilocular cysts Hyperechoic lesions
Kim et al., 2018 [23]	Ill-defined low attenuating head mass	Locally advanced mass in the head and an enhancing mass in the tail	-	-	-
Mori et al., 2018 [24]	-	-	-	-	-
Huang et al., 2018 [25]	Two hypodense masses	-	-	-	Hypoechoic solid masses Atrophic pp between the two masses
McGregor et al., 2018 [26]	Well-circumscribed, enhanced lesion with cystic components in the head Heterogeneously enhanced hypervascular lesion in the tail	-	-	-	Heterogenous hypoechoic lesion with irregular margin
Sugiura et al., 2019 [27]	MPD dilation Heterogeneously enhancing lesion	Multilocular cyst without enhancement Heterogeneously enhancing lesion	Cyst in the tail MPD dilation	-	-
Ohno et al., 2020 [28]	Mass in the head that had invaded the right side of the SMV and in the tail	-	-	-	-
Liu et al., 2020 [29]	-	Multilocular cystic mass in the tail	-	-	-
Schlanger et al., 2021 [6]	-	Polycystic head tumor MPD dilation	-	-	Cystic lesion at the level of the uncinate process
Aaquist et al., 2021 [31]	Hypodense lesion in the head				
Paramythiotis et al., 2022 [32]	Two ill-defined hypoenhancing lesions MPD dilation and obstruction Abutment of the SMV	-	Ill-defined hypointense lesion in the head MPD dilation IPMN in the pancreatic parenchyma	-	Hypoechoic masses in the head and tail
Aloraini et al., 2023 [33]	Head heterogeneous necrotic lesion MPD dilation	Localized lesion in the head and dilatation in the intra- and extrahepatic biliary trees	-	-	-
	Dilated intra- and extrahepatic biliary trees with multiple gallstones and no vascular invasion	MPD dilation Atrophy and mild intra- and extrahepatic biliary duct dilatation	MPD dilation Atrophy and mild intra- and extrahepatic biliary duct dilatation	-	-

**Table 7:** This table outlines clinical and treatment details for patients with pancreatic tumors. The columns include the intervention, specifying the surgical procedure performed, such as total or distal PE (pancreatectomy) or Pp PD (pylorus-preserving pancreatoduodenectomy) or MSPP (middle segment preserving pancreatectomy); stage, indicating the tumor’s clinical or pathological stage; chemotherapy, detailing the use of chemotherapy in treatment; recurrence, noting whether the tumor recurred post-treatment; postoperative treatment, describing any additional therapies administered after surgery; and survival (months), representing the duration of patient survival in months following treatment.

Study ID	Intervention	Stage	Chemotherapy	Recurrence	Postoperative Treatment	Survival (Months)
Posniak et al., 1990 [11]	-	-	-	-	-	-
Montag et al., 1990 [12]	Total PE	-	-	-	-	Death
	Total PE	-	-	-	-	9 (Death)
Nodell et al., 1993 [13]	Pp Whipple	-	-	-	-	-
Siassi et al., 1999 [14]	MSPP	I	-	No	Glucose control	12
Nitta et al., 2008 [34]	Whipple	-	-	-	-	-
Izumi et al., 2009 [16]	Pp PD	-	S-1	Yes		6
Fujimori et al., 2010 [17]	Total PE	IIB	Gemcitabine	No	Insulin	12
Mori et al., 2010 [18]	Total PE	-	Gemcitabine	No	Glucose control	6
Goong et al., 2015 [19]	Patient Refused Surgery	IIB	Chemoradiotherapy	-	-	-
De Silva et al., 2017 [20]	Total PE	IIA	-	-	Insulin and oral penicillin	-
Serafini et al., 2017 [21]	Total PE	-	Refused	No		8
Wang et al., 2018 [22]	Whipple	-	-	No		12
Kim et al., 2018 [23]	Radical PE (4 years after Whipple)	-	Chemoradiotherapy	No		60
Mori et al., 2018 [24]	Total PE	IB	-	-		-
Huang et al., 2018 [25]	No	-	-	-		-
McGregor et al., 2018 [26]	Total PE	-	FOLFIRINOX (n)	No	Ιnsulin pump	39
Sugiura et al., 2019 [27]	Palliative chemotherapy	IV	-	-		-
Ohno et al., 2020 [28]	Total PE	-	Gemcitabine and nab-paclitaxel	Yes		10 (Death)
Liu et al., 2020 [29]	Total PE	-	Gemcitabine	No		10
Nitta et al., 2020 [15]	MSPP	IB and IIB	S-1	No	DDP-4	9
Ohike et al., 2020 [7]	Distal PE	IA and IIA	-	Yes		65 (Death)
Fujita et al., 2020 [30]	PE	IIB	-	No		53
	PE	IIB	-	Yes		48
	PE	IIB	-	No		50 (Death)
	PE	IIB	-	Yes		44 (Death)
	PE	III	Chemoradiotherapy	Yes		27 (Death)
	PE	III	-	Yes		35 (Death)
	PE	III	-	Yes		15 (Death)
Schlanger et al., 2021 [6]	Total PE	IA	Adjuvant	No		36
Aaquist et al., 2021 [31]	Whipple	IA	-	-		-
Paramythiotis et al., 2022 [32]	Total PE	IB and IIB	Gemcitabine	No	Anticoagulants (due to DVT)	5
Aloraini et al., 2023 [33]	Total PE	III	Gemcitabine and Cisplatin	No		8 (Death)
	Total PE	III	Refused	No		6 (Death)

## Data Availability

No new data were created in this study. Data sharing is not applicable to this article.
